# Analytical Report on the Output of “Iranian Journal of Public Health” During 2014–2017

**Published:** 2018-02

**Authors:** Dariush D FARHUD

According to annual review of Iran J Public Health, in this issue a brief review is presented in terms of analyzing the number and kind of papers submitted and published during the year 2017. Besides, this editorial will compare the trend of whole publication process during 2014–2017.

**Fig. 1:**
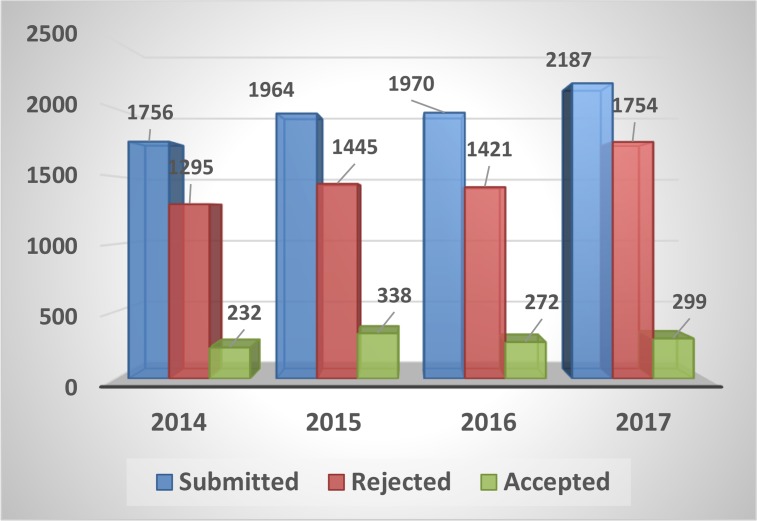
Total number of articles submitted during 2014–17 in the context of the frequency of submission, rejection and acceptance rate

The total number of manuscripts received during 2017 was 2187 from 53 countries. Of course, only the country of corresponding author was considered, so altogether much more countries we had in the panel. Again, Iran had the highest rate of submission, followed by China and Turkey (Table 1). Fig. 1 presents total number of articles published during 2014–17 in the context of the frequency of submission, rejection and acceptance rate.

**Table 1:** Frequency of manuscripts received by Iran J Public Health during 2017 in terms of the frequency of submission, rejection and acceptance rate

**NO.**	**Country**	**Submitted**	**Rejected**	**Accepted**
**1**	**Algeria**	2	1	1
**2**	**Australia**	1	1	0
**3**	**Bangladesh**	7	7	0
**4**	**Bosnia**	1	1	0
**5**	**Brazil**	14	11	3
**6**	**Bulgaria**	2	2	0
**7**	**Canada**	2	1	1
**8**	**Czech**	4	2	1
**9**	**China**	273	199	52
**10**	**Cyprus**	5	5	0
**11**	**Egypt**	8	5	1
**12**	**Ethiopia**	4	3	1
**13**	**Gaza**	2	2	0
**14**	**Georgia**	1	1	0
**15**	**Ghana**	1	1	0
**16**	**Hungary**	1	1	0
**17**	**India**	25	24	1
**18**	**Indonesia**	23	17	3
**19**	**Iran**	1163	937	146
**20**	**Iraq**	10	9	0
**21**	**Italy**	5	3	2
**22**	**Jordan**	14	12	1
**23**	**Kazakhstan**	23	15	7
**24**	**Korea**	91	53	30
**25**	**Kuwait**	1	1	0
**26**	**Libya**	2	2	0
**27**	**Macedonia**	4	1	3
**28**	**Malaysia**	41	34	4
**29**	**Mexico**	4	2	1
**30**	**Moldova**	1	1	0
**31**	**Morocco**	7	5	2
**32**	**Nepal**	1	1	0
**33**	**Nigeria**	8	8	0
**34**	**Pakistan**	104	97	4
**35**	**Palestinian**	1	1	0
**36**	**Poland**	16	15	1
**37**	**Romania**	17	11	5
**38**	**Saudi Arabia**	18	16	0
**39**	**Senegal**	1	1	0
**40**	**Serbia and Montenegro**	12	9	3
**41**	**Slovakia**	4	0	4
**42**	**Slovenia**	3	2	1
**43**	**South Africa**	11	8	3
**44**	**Spain**	2	2	0
**45**	**Sri Lanka**	4	4	0
**46**	**Sudan**	1	1	0
**47**	**Taiwan**	10	9	0
**48**	**Thailand**	9	5	2
**49**	**Tunisia**	32	25	7
**50**	**Turkey**	183	175	6
**51**	**Ukraine**	3	2	1
**52**	**United States**	1	1	0
**53**	**Vietnam**	4	2	2
**Total**	**-**	**2187**	**1754**	**299**

Out of total submission of 2187 articles during 2017, 1754 articles were rejected after initial in-house evaluation or later peer review. Therefore the acceptance rate for this year was 13.6%.

As previous years, some cases of plagiarism were detected and were treated according to the policy of the journal. Normally, authors of minor cases of plagiarism are given a chance to amend their manuscripts precisely but major cases are rejected. Unfortunately, the dilemma of plagiarism still occurs in a portion of submitted articles mostly sent from non-English native countries.

**Fig. 2:**
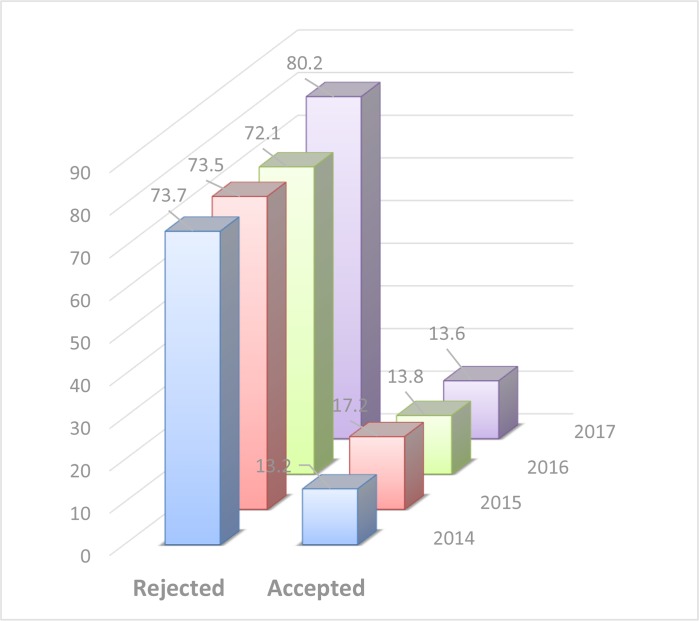
Total number of articles published during 2014–17 in terms of the percent of acceptance and rejection rate

The Journal follows a policy of in-house evaluation followed by double blind peer review system. As for foreigner authors we try to exert an open peer review system. The reasons for rejecting a manuscript during in-house evaluation are various but the most important cases are out of scope cases, poor outcome, local studies, clinical contents etc. Fig. 2, demonstrates the total number of articles published during 2014–2017 in terms of the percent of acceptance and rejection rate. It is worth mentioning that some manuscripts submitted during 2017, are still in the process of peer review so we have no idea of their destination. This may cause some problems in reporting exact data. However, the rejection rate in 2017 was 80.2%.

A critical point is that due to high rate of receiving articles from different countries, up to now nearly 260 articles are in the queue of lay outing and we have no choice but to delay the date of publication.

**Fig. 3:**
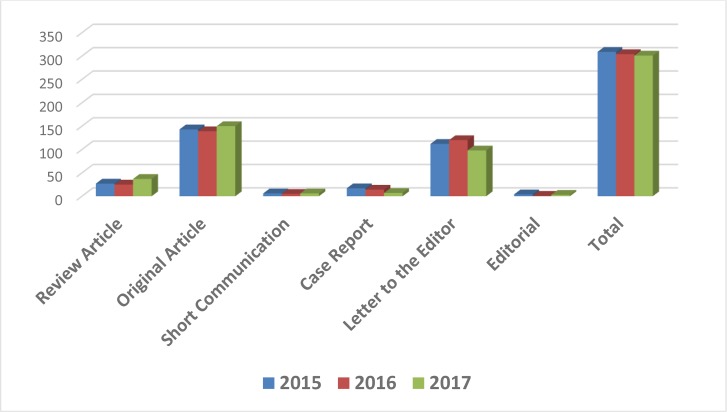
Total number of articles during 2014–17 based on the type of published papers

The types of articles published during 2014–2017 are shown in Fig. 3. Accordingly, Original Articles had the highest rate of publication during the last three years.

Due to high flow of submitted manuscripts, in many cases, the authors were requested to change the format of “Original Article” to “Letter to the Editor”, which of course the merit of both formats remains the same.

According to http://www.scimagojr.com/, the H index of the journal is 23. Besides SCOPUS has reported the Site Score of the journal as 0.85 for 2016. According SJR 2016 is 0.372 in addition to SNIP 2016 as 0.613.

**Fig. 4:**
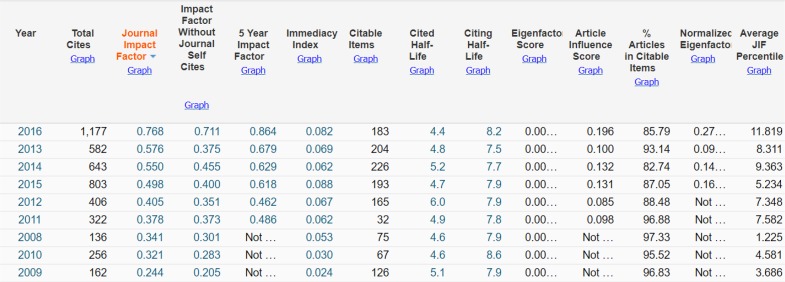
Key indicators announced by Clarivate Analytics (ISI) including Impact factor of IJPH during 2009–16

Fig. 4 shows some key indicators reported by Clarivate Analytics (ISI) during 2009–16 including Impact Factor for the year 2016 as 0.768.

